# Self-Powered Electrical Impulse Chemotherapy for Oral Squamous Cell Carcinoma

**DOI:** 10.3390/ma15062060

**Published:** 2022-03-10

**Authors:** Chaochao Zhao, Yuan Yang, Xi Cui, Yizhu Shan, Jiangtao Xue, Dongjie Jiang, Jinyan Sun, Na Li, Zhou Li, Anping Yang

**Affiliations:** 1 Department of Biomedical Engineering, School of Medicine, Foshan University, Foshan 528225, China; zhaochaochao@fosu.edu.cn (C.Z.); jinyansun@fosu.edu.cn (J.S.); lina1102@fosu.edu.cn (N.L.); 2 Beijing Key Laboratory of Micro-Nano Energy and Sensor, Beijing Institute of Nanoenergy and Nanosystems, Chinese Academy of Sciences, Beijing 101400, China; yangyuan@binn.cas.cn (Y.Y.); cuixi@binn.cas.cn (X.C.); shanyizhu@binn.cas.cn (Y.S.); xuejiangtao@binn.cas.cn (J.X.); jiangdongjie@binn.cas.cn (D.J.); zli@binn.cas.cn (Z.L.); 3 School of Nanoscience and Technology, University of Chinese Academy of Sciences, Beijing 101400, China; 4 School of Life Science, Beijing Institute of Technology, Beijing 100081, China

**Keywords:** oral squamous cell carcinoma, electrical impulse chemotherapy, self-powered, triboelectric nanogenerator

## Abstract

Oral squamous cell carcinoma (OSCC) is a common oral cancer of the head and neck, which causes tremendous physical and mental pain to people. Traditional chemotherapy usually results in drug resistance and side effects, affecting the therapy process. In this study, a self-powered electrical impulse chemotherapy (EIC) method based on a portable triboelectric nanogenerator (TENG) was established for OSCC therapy. A common chemotherapeutic drug, doxorubicin (DOX), was used in the experiment. The TENG designed with zigzag structure had a small size of 6 cm × 6 cm, which could controllably generate the fixed output of 200 V, 400 V and 600 V. The electrical impulses generated by the TENG increased the cell endocytosis of DOX remarkably. Besides, a simply and ingeniously designed microneedle electrode increased the intensity of electric field (EF) between two adjacent microneedle tips compared with the most used planar interdigital electrode at the same height, which was more suitable for three-dimensional (3D) cells or tissues. Based on the TENG, microneedle electrode and DOX, the self-powered EIC system demonstrated a maximal apoptotic cell ratio of 22.47% and a minimum relative 3D multicellular tumor sphere (MCTS) volume of 160% with the drug dosage of 1 μg mL^−1^.

## 1. Introduction

Oral cancer accounts for about 3% of all malignant tumors, among which oral squamous cell carcinoma (OSCC) is the most common [1,2,3]. The factors, such as alcohol, smoking, human papillomavirus infection, diet and genes, can lead to abnormal gene expression, thus promoting the development of OSCC [4]. There are 300,000 cases of OSCC in the world every year [5], and the incidence is increasing year by year, especially among young people [6]. OSCC patients usually have distant metastasis at the initial diagnosis, thus missing the optimal therapeutic period. In this regard, the 5-year survival rate is only about 50% [7]. The disease causes great pain to the patients physically and mentally, which needs effective treatment. Clinically, the main therapy methods are chemotherapy, radiotherapy and surgery, among which chemotherapy is most widely used, especially for the advanced OSCC patients. The body can produce strong drug resistance to the common chemotherapy drugs including doxorubicin (DOX), platinum drugs, paclitaxel, etc. Meanwhile, large dosages, high toxicity and low efficiency have become a great interference in the process of chemotherapy [8].

Pulsed electric fields can boost the delivery of drugs into tumor cells, and is called electrical impulse chemotherapy (EIC) [9]. In 1991, Mir and his colleagues at the Gustave Roussy Institute in France conducted the world’s first clinical trial of EIC, using bleomycin and electrical impulses for the treatment of recurrent OSCC [10]. Subsequently, clinical trials were carried out in the United States, France, Germany, Ireland, Australia, Austria, Yugoslavia and other countries [11,12,13,14]. Reintgen et al. observed that basal cell carcinoma had an obvious response to EIC which could greatly reduce the dose of chemotherapy drugs [15]. In 2021, Sundararajan et al. found that electrical impulses could increase the cell uptake of curcumin and thus enhanced the drug toxicity to cancer cells [16]. EIC has unique advantages in cancer therapy, which can reach the targeted tissue and make it easier to operate [17,18]. However, traditional electrical impulse devices usually have the inconvenience of huge mass, poor portability and low safety [19]. Moreover, the electrode materials, encapsulation quality and rigid structure strongly affect the normal tissue and even produce a great threat to the activity of human life, seriously hindering the development of this technology in oral cancer treatment and clinical application.

The invention of the self-powered energy collecting devices represented by triboelectric nanogenerators (TENG) provide a feasible solution to this problem [20,21,22,23,24]. The TENG was first proposed by Zhong Lin Wang in 2012 that could convert the mechanical energy in the environment into electrical energy based on the coupling of triboelectrification effect and electrostatic induction effect [25]. The TENGs have the characteristics of high voltage up to 1000 volts, low current, good safety, simple preparation process and wide material selectivity [26,27,28,29,30]. Biological life activities such as breathing, heartbeat, limb action, etc. contain rich mechanical energy, which can drive the TENGs to produce electrical output [31,32,33,34,35]. Our previous work developed an implantable magnet TENG which could generate a voltage of 70 V. Combined with the drug loaded red blood cell, the drug delivery system (DDS) had excellent anti-tumor ability with cervical cancer [36]. Nonetheless, the introduced RBC from the living body significantly increased the complexity, long-term stability as well as price of the DDS.

In this work, we demonstrated a self-powered EIC method for OSSC therapy based on the TENG. The prepared TENG could precisely control the voltage output. Besides, a microneedle electrode was designed to provide higher electric field (EF) applied to the three-dimensional (3D) cells or tissues. The self-powered EIC could not only kill two-dimensional (2D) carcinoma cells, but also inhibit the growth of 3D multicellular tumor sphere (MCTS), demonstrating an outstanding anti-tumor efficacy. This study was intended to develop a high-performance and portable electrical impulse source to realize EIC for oral cancer, which has great scientific and clinical significance.

## 2. Materials and Methods

### 2.1. TENG Fabrication

The working strategy of the designed TENG was based on the vertical contact-separation mode. An aluminum (Al) sheet with a microstructure and a polytetrafluoroethylene (PTFE) membrane with a nanopillar structure were used as the two triboelectric layers. The Al sheet was polished with sandpaper three times to increase the friction area. The surface of the PTEF membrane was also treated with inductively coupled plasma (ICP) reactive ion etching to form the nanostructure. Before etching, the PTFE was covered by an aurum (Au) film with a few nanometers as a mask. Then the gases of CF_4_, O_2_ and Ar with the corresponding flow rates of 30, 15 and 10 sccm were mixed in the main chamber. The power source of 400 W was used in the etching process. Next, a cuprum (Cu) film was magnetron sputtered on the PTFE membrane as the back electrode. After the two friction layers were connected with leading wires, they were fixed on a resilient polyethylene terephthalate (PET) substrate with a zigzag structure. The electric signals were measured by a current amplifier from Stanford Research Systems (SR570) (Stanford Research Systems, Sunnyvale, CA, USA) and a Keithley electrometer system (6517b) (Tektronix, Beaverton, OR, USA).

### 2.2. 2D Stimulation Device Preparation

A piece of flexible PET substrate was cleaned by acetone, ethyl alcohol and deionized water, respectively. Then, after the SUN-115P photoresist (Suntific Materials, Weifang, China) was spin-coated on the PET substrate, it was baked at 90 °C for 60 s. The ultraviolet exposure progress was carried out under a high-resolution mask for 12 s. After that, the PET was baked at 90 °C for 60 s and immersed in the photoresist developer for 15 s. Next, a Cu electrode on the PET substrate was magnetron sputtered on the front side of the PET substrate. After excess Cu was removed with a moderate stripping liquid, the soft electrode was finally prepared. For further encapsulation and insulation, a thin PDMS film was spin-coated on the surface. Finally, a biocompatible poly lactic acid (PLA) well was pasted on the surface of the electrode for cell culture.

### 2.3. Cell Culture

Tca-8113 cells bought from Mingzhou Biotechnology Company (Ningbo, China) were incubated with 1640 medium containing 100 g mL^−1^ streptomycin, 100 g mL^−1^ penicillin and 10% (*v*/*v*) FBS in a cell incubator that kept the temperature at 37 °C. The cells were passaged every two days. For 3D MCTS forming, 50 μL agarose solution was coated in each well of a 96-well plate, and then 200 μL dispersed Tca-8113 cells (count: 2500) in 1640 media were seeded inside the wells. After four days of growth, the diameter of the MCTSs reached about 400 μm.

### 2.4. 2D Cell Toxicity

The cell toxicity was measured with a 3-(4,5-dimethylthiazol-2-yl)-2,5-diphenyltetrazolium bromide (MTT) assay. Briefly, 100 μL MTT solution (5 mg mL^−1^) was added into each well of the 96-well plate and incubated with the cells for 4 h in a cell incubator. Then after removing the supernatant, 200 μL dimethyl sulfoxide (DMSO) (Aladdin, Shanghai, China) was added for dissolving the generated formazan. The absorbance was measured with a spectrophotometer at the wavelength of 570 nm. The live and dead cell experiment was implemented using a live and dead cell staining kit and the fluorescent images were acquired by a fluorescence microscope (Olympus IX 71) (Olympus, Tokyo, Japan).

### 2.5. Microneedle Electrode Preparation

Firstly, a four-microneedle polydimethylsiloxane (PDMS) mold was prepared using a high precision laser cutting system. Then a PLA microneedle paster was produced using hot pressure technology and a Cu film was coated on the surface as an electrode using a magnetron sputtering method. After the newly formed Cu electrode was separated by a sharp knife, the surface of the electrode was encapsulated by parylene C. Thereafter, the microneedle electrodes were tailored into proper sizes.

### 2.6. Apoptosis Assay of the MCTS

When the dimeter of the MCTSs reached about 400 μm, DOX with the concentration of 1 μg mL^−1^ was added on Day 0. The electrical impulses generated by the TENG were applied to the cells and kept for 1 h every day using the microneedle electrodes. On Day 3, the terminal-deoxynucleoitidyl transferase mediated nick and labeling (TUNEL) experiments were carried out using the cell death detection kit (Merck Millipore, Darmstadt, Germany).

### 2.7. Growth Inhibition of the MCTS

The culture media of the MCTSs were half replaced every two days. The bright field images of the MCTSs were obtained by an optical microscope and digital camera system every day, and the sizes of the MCTSs were recorded by the diameter (d). The final volumes of the MCTSs were calculated by the spherical volume equation. Each group had four parallel tests, and the data were obtained by the mean volumes.

## 3. Results and Discussion

### 3.1. Fabrication and Characterization of the TENG

**Figure 1:**
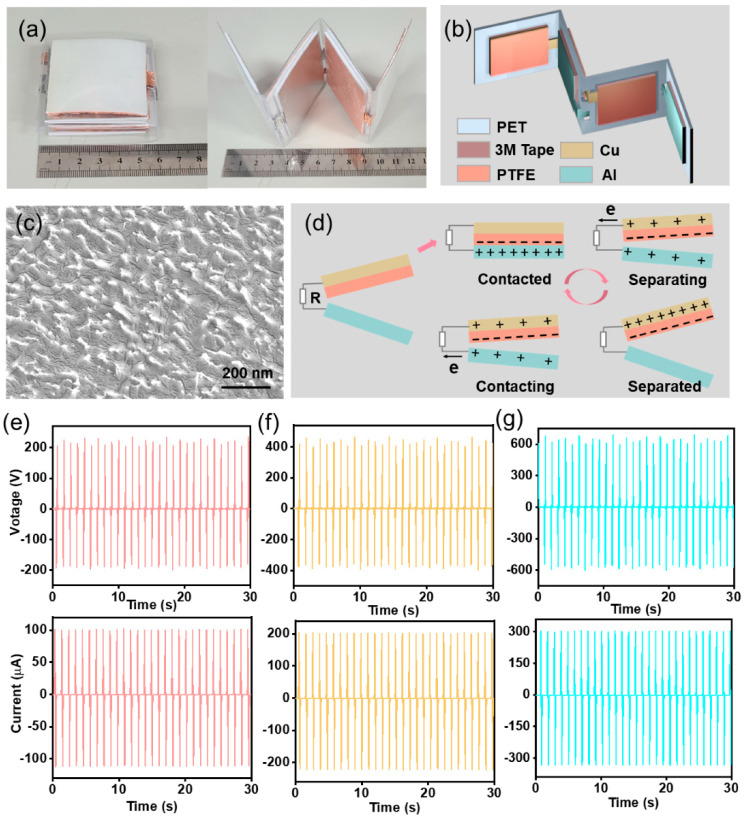
The working principle and output performance of the output controllable triboelectric nanogenerator (TENG). (**a**) The physical pictures of the TENG. (**b**) The schematic picture of the TENG. (**c**) The scanning electron microscope (SEM) image of the polytetrafluoroethylene (PTFE) surface. (**d**) The working principle of the fabricated TENG. (**e**) The *V*_oc_ and *I*_sc_ when two polyethylene terephthalate (PET) layers were working. (**f**) The *V*_oc_ and *I*_sc_ when three PET layers were working. (**g**) The *V*_oc_ and *I*_sc_ when four PET layers were working.

The working principle of the TENG in this experiment was based on the vertical contact-separation mode due to simple structure and high instantaneous power density. The two friction layers, an Al sheet and a PTFE membrane, were fixed on a zigzag PET substrate with 3M tape (Figure 1a). The structure diagram of the TENG was shown in Figure 1b. The ICP etching technique was used to form nanostructures on the PTFE surface to enlarge the output of the TENG (Figure 1c). In addition, the surface of Al foil was polished by an abrasive paper for three times (Figure S1). A Cu film with a thickness of about 200 nm was magnetron sputtered on the back of the PTFE membrane as electrode. Based on the coupling of contact electrification and electrostatic induction, the detailed working principle and COMSOL stimulation of the TENG were illustrated in Figure 1d and Figure S2. Under periodic contact and separation of the two triboelectric layers, electrons were driven back and forth through the external circuit. The nanostructures on the surface of the PTFE film and Al sheet could increase their friction area and then generate more electric charges on the contact surface. Besides, the back 3M tape could guarantee the complete contact of the two friction layers and the high resistance of the PET substrate could realize the entire separation after contact. Moreover, the zigzag structure could both efficiently reduce the size of the whole device and precisely generate fixed maximum output. When two layers were contacted and separated, the TENG could generate an open-circuit voltage (*V*_oc_) of about 200 V, a short-circuit current (*I*_sc_) of about 100 μA (Figure 1e) and a transferred charge (*Q*_sc_) of about 200 nC (Figure S3a). As periodically pressing three layers, the TENG could output an open-circuit voltage (*V*_oc_) of about 400 V, a short-circuit current (*I*_sc_) of about 200 μA (Figure 1f) and a transferred charge (*Q*_sc_) of about 400 nC (Figure S3b). While four layers were working simultaneously, the TENG could produce an open-circuit voltage (*V*_oc_) of about 600 V, a short-circuit current (*I*_sc_) of about 300 μA (Figure 1g) and a transferred charge (*Q*_sc_) of about 300 nC (Figure S3c). If the TENG ran for an hour at 1 Hz, it would generate the charges of about 2.16 mC. However, only 1.08 mC could be stored in a capacitor of 100 μF (Figure S4). After 10^5^ trials, the output of the TENG kept stable (Figure S5). For proper working of the TENG, the power of the TENG was further tested. The maximum power was 48 mW when the load was about 2 × 10^7^ Ω (Figure S6). Besides, the voltage of 600 V was detected between the microneedle electrodes. This portable output and controllable TENG was convenient for providing constant electrical impulses and studying the biomedical effect of electric stimulation, and stood a good chance to become a potent tool in clinic.

### 3.2. TENG Controlled EIC to Treat 2D Tca-8113 Cells

**Figure 2:**
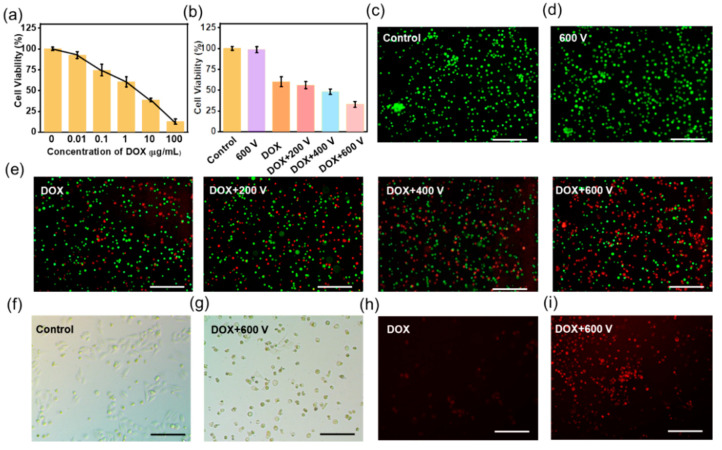
The self-powered electrical impulse chemotherapy (EIC) to treat 2D Tca-8113 cells. (**a**) The 3-(4,5-dimethylthiazol-2-yl)-2,5-diphenyltetrazolium bromide (MTT) assay of different doxorubicin (DOX) concentrations. (**b**) The cell viabilities of Tca-8113 cells in the control, 600 V, DOX + 200 V, DOX + 400 V and DOX + 600 V groups. (**c**–**e**) The live and dead images of Tca-8113 cells in different groups. The microscope photos of the control group (**f**) and the DOX + 600 V group (**g**). The fluorescence microscope photographs of the DOX group (**h**) and the DOX + 600 V group. Scale bar: 100 μm (**i**).

The self-powered stimulation device for EIC was integrated with a TENG and an interdigitated electrode. A Flexible PET sheet was chosen as the substrate, upon which Au electrodes with gaps of 100 μm and widths of 100 μm were fabricated with a photolithography technique and magnetron sputtering process. Further, the surface of the Au electrodes was covered by an ultra-thin PDMS film for insulation, which could reduce the influence of current. When the TENG was working, the cells on the PDMS surface could be stimulated by the electrical impulses. Before EIC experiment, the toxicity of DOX was tested by the MTT assay and the value of half maximal inhibitory concentration (IC 50) was between 1 μg mL^−1^ and 10 μg mL^−1^ (~5.3 μg mL^−1^), as shown in Figure 2a. Hence, the concentration of 1 μg mL^−1^ was used in the following experiments. One hour after the DOX was added, the Tca-8113 cells were stimulated by the electrical impulses for another one hour every day. Thereafter the cell viabilities were evaluated by the MTT assay. The result showed that 600 V stimulation alone didn’t affect the viability of Tca-8113 cells. The viability of tumor cells in 1 μg mL^−1^ DOX was decreased to 60.1%. When combined with electrical impulses, the group of DOX plus 200 V (DOX + 200 V) and the group of DOX plus 400 V (DOX + 400 V) had a slight lower viability of 56.2% and 48.4%, respectively. It was worth noting that the DOX + 600 V group had much reduced cell viability of 32.8% (Figure 2b). Moreover, the live and dead analysis pictures of Tca-8113 cells were shown in Figure 2c–e, which were consistent with the MTT assay. The output of 200 V generated an EF below 1 Kv/cm, which was not enough for the self-powered EIC process. When the output was 400 V, an EF of about 2 Kv/cm could be produced, which increased the cell toxicity to a certain extent. Significantly, as the voltage of 600 V was applied, the EF of above 3 Kv/cm killed about two-thirds of the tumor cells, which was effective in the EIC. The above results suggested that the self-powered EIC had increased killing ability of oral cancer cells in a 2D culture model.

The normal morphology of Tca-8113 cells in the control group was shown in Figure 2f. However, the cells in the DOX + 600 V group shrank and tended to be round shape, suggesting that most cells were apoptosis (Figure 2g). To study the mechanism of the self-powered EIC, a fluorescence microscope was employed to observe the change after the electrical impulses. The DOX used in the experiment had a fluorescence emission peak of 590 nm (Figure S7), which was shown inside a small number of Tca-8113 cells in the DOX group (red, Figure 2h). While the DOX was phagocytized by most cells in the DOX + 600 V group, thus increasing the cytotoxicity (Figure 2i).

### 3.3. Microneedle Electrode Fabrication

**Figure 3:**
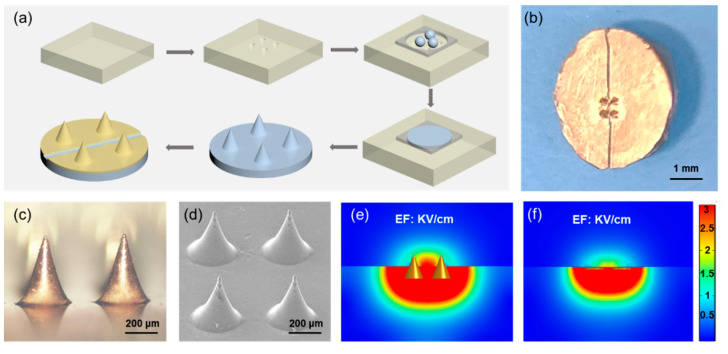
The fabrication of the microneedle electrode. (**a**) The manufacturing process of the microneedle electrode. (**b**) The digital picture of the fabricated microneedle electrode. (**c**) The optical microscope image of the microneedles. (**d**) The SEM image of the microneedles. The finite element analysis of the microneedle electrode (**e**) and the planar electrode (**f**) with the voltage of 600 V.

Plane interdigital electrode were most used in the 2D in vitro cell stimulation studies [37,38,39], but they had little influence on the inner part of 3D cells or tissues because of the extremely low EF away from the electrode position. For much higher EF and wider applicability for 3D cells or tissues, a microneedle electrode was designed and prepared. The fabrication progress was very simple and easy to operate, shown in Figure 3a. Briefly, a piece of PDMS mold was produced by a laser cutting system, which contained four microneedle holes. The PLA microneedle paste was prepared using hot pressure method and a Cu film was coated on the surface using magnetron sputtering method. After the Cu electrode was separated by a sharp knife (Figure 3b), a parylene C film was evaporated on the surface for insulation. The morphology of the microneedles was present in Figure 3c obtained by an optical microscope and Figure 3d acquired by a scanning electron microscope (SEM). The stiffness of the PLA microneedles had already been tested by the animal experiment [40]. From the finite element analysis, when the applied voltage was 600 V, the EF of microneedle electrode was above 3 kV/cm (Figure 3e). The EF intensity the microneedle electrode between two adjacent microneedle tips was much higher than that of the most used planar interdigital electrode at the same height (Figure 3f), which was more suitable for 3D MCTS systems or tissues. The same results could be obtained when the applied voltages were 200 V (Figure S8) and 400 V (Figure S9).

### 3.4. The Self-Powered EIC to Treat 3D MCTS

**Figure 4:**
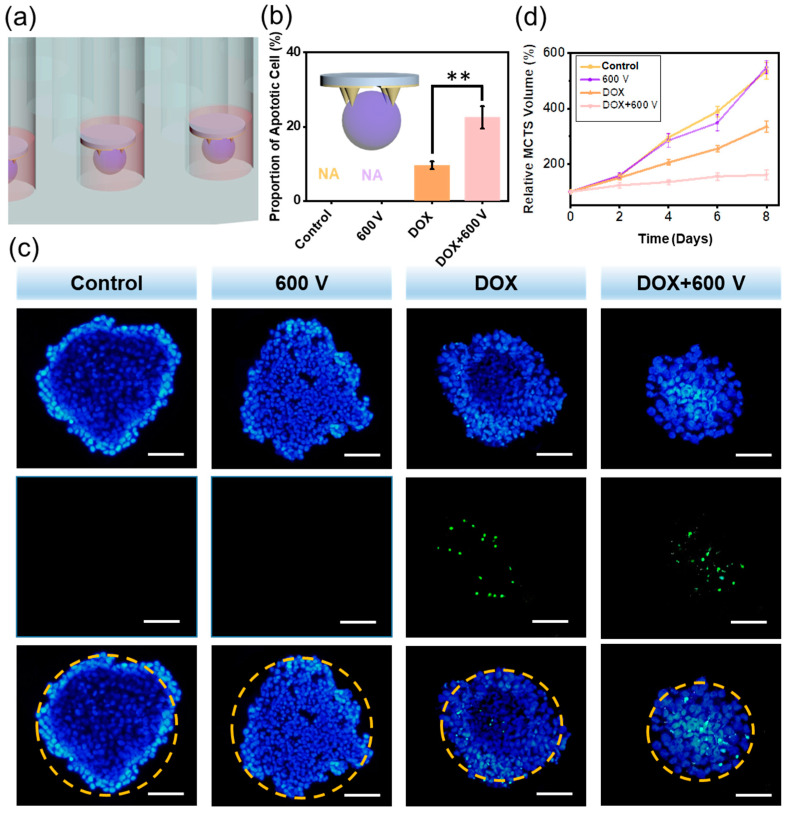
The self-powered EIC to treat 3D multicellular tumor sphere (MCTS) system. (**a**) The schematic diagram of the microneedle electrodes and MCTSs. (**b**) The proportions of apoptotic cell calculated in the TUNEL experiment, *p* values: ** *p* < 0.01. (**c**) The TUNEL images in the control, 600 V, DOX and DOX + 600 V groups. Scale bar: 100 μm. (**d**) The volume growth of Tca-8113 MCTSs in different groups (*n* = 4).

The 3D MCTS culture system could reflect the actual situation inside tumor environment to a great extent. Besides, the microneedle electrode exhibited much higher possibility to be used for 3D cells and tissues. In this regard, there was an urgent demand to study the self-powered EIC system in a 3D evaluation system. An agarose 3D culture system was used in the next experiment, which was exhibited in Figure 4a,b (insert).

After four days of growth, the diameter of the MCTSs reached about 400 μm, setting as on Day 0 (Figure S10). Then the DOX was added and electrical impulses were applied to the MCTSs for 1 h every day. The therapy effect of self-powered EIC was demonstrated using TUNEL experiment on Day 3, which showed nonuniform cytotoxicity on the MCTSs (Figure 4c). The control group and the 600 V group didn’t affect the survival of cancer cells, just as expected. We found that compared with the DOX group, the DOX + 600 V group showed more apoptotic cells, because the electrical impulses were delivered to the MCTSs by the microneedle electrode, thus enhancing the DOX endocytosis by Tca-8113 cells. Moreover, the diameters of the MCTS in the DOX + 600 V group were much smaller than that in the DOX group. The proportions of apoptosis were calculated, during which the DOX + 600 V group had the maximum ratio of 22.47% (Figure 4b). From Figure 4d, the control group and the 600 V group had uncontrollable volume growth of the MCTSs, and the DOX alone was difficult to completely inhibit the tumor growth. While the MCTSs in the DOX + 600 V group rarely grew at the very early stage and reached only 1.6 times of their original volumes after 8 days, demonstrating the most satisfactory result for antitumor therapy.

## 4. Conclusions

OSCC, as a common malignant cancer of head and neck, is a severe threat to people’s health. Among various clinical treatments, chemotherapy is most widely used. However, the chemotherapeutic drugs usually do harm to the kidney, stomach, intestines, heart, and other organs. In this regard, reduced dosage but with high therapeutic effect is the goal we pursue. In this work, we developed a portable TENG which had a small size of 6 cm × 6 cm and high output up to 600 V. The TENG could controllably generate fixed output of 200 V, 400 V and 600 V, which was important for biomedical applications. With the TENG acting as the electrical impulse source and the microneedle electrodes, this self-powered EIC system gained tumor cell killing rate of 51.6% in the 2D system and 22.47% in the 3D system with reduced drug concentration of 1 μg mL^−1^. Besides, the MCTSs in the self-powered EIC system grew slowly, and after eight days the volumes were only 1.6 times of the initial.

In summary, we successfully established a self-powered EIC for OSCC therapy. When the TENGs were working, the generated electric pulses were delivered to the microneedle electrodes. Then the altering high EF could increase the tumor cell endocytosis of DOX, thus enhancing the antitumor effect. This self-powered EIC demonstrated a distinguished therapeutic effect of cancer with very low DOX dosage, which was highly promising to be used in the clinical medicine.

## Data Availability

Data is contained within the article.

## Supplementary Materials

The following supporting information can be downloaded at: https://www.mdpi.com/article/10.3390/ma15062060/s1, Figure S1: The surface microstructure of the Al sheet acquired by an optical microscope; Figure S2: The TENG’s electrical distribution of charges with COMSOL software; Figure S3: The *Q*_sc_ when two, three and four PET layers working; Figure S4: The charging curve of the TENG; Figure S5: The stability test of the TENG; Figure S6: The power measurement of the TENG; Figure S7: The fluorescence emission curve of the DOX; Figure S8: The finite element analysis of the microneedle electrode and planar electrode with the voltage of 200 V; Figure S9: The finite element analysis of the microneedle electrode and planar electrode with the voltage of 400 V; Figure S10: The optical microscope image of the MCTS on Day 0.
