# Supplementary material for: Self-Powered Electrical Impulse Chemotherapy for Oral Squamous Cell Carcinoma
Source: Materials (Basel). 2022 Mar 10;15(6):2060. doi: 10.3390/ma15062060 (PMC8954269; doi:10.3390/ma15062060)
Supplement: Supplementary file 1 [file materials-15-02060-s001.zip › materials-1570004-supplementary.pdf]

Supplementary Materials

# Self-Powered Electrical Impulse Chemotherapy for Oral Squamous Cell Carcinoma

**Chaochao Zhao [1,2], Yuan Yang [2,3], Xi Cui [2,3], Yizhu Shan [2,3], Jiangtao Xue [2,4], Dongjie Jiang [2,3], Jinyan Sun [1], Na Li [1], Zhou Li [2,3] and Anping Yang [1,*]**

1 Department of Biomedical Engineering, School of Medicine, Foshan University, Foshan 528225, China; zhaochaochao@fosu.edu.cn (C.Z.); jinyansun@fosu.edu.cn (J.S.); lina1102@fosu.edu.cn (N.L.)
2 Beijing Key Laboratory of Micro-Nano Energy and Sensor, Beijing Institute of Nanoenergy and Nanosystems, Chinese Academy of Sciences, Beijing 101400, China; yangyuan@binn.cas.cn (Y.Y.); cuixi@binn.cas.cn (X.C.); shanyizhu@binn.cas.cn (Y.S.); xuejiangtao@binn.cas.cn (J.X.); jiangdongjie@binn.cas.cn (D.J.); zli@binn.cas.cn (Z.L.)
3 School of Nanoscience and Technology, University of Chinese Academy of Sciences, Beijing 101400, China
4 School of Life Science, Beijing Institute of Technology, Beijing 100081, China
* Correspondence: foshanyap@fosu.edu.cn

Supplementary Figures S1–10.

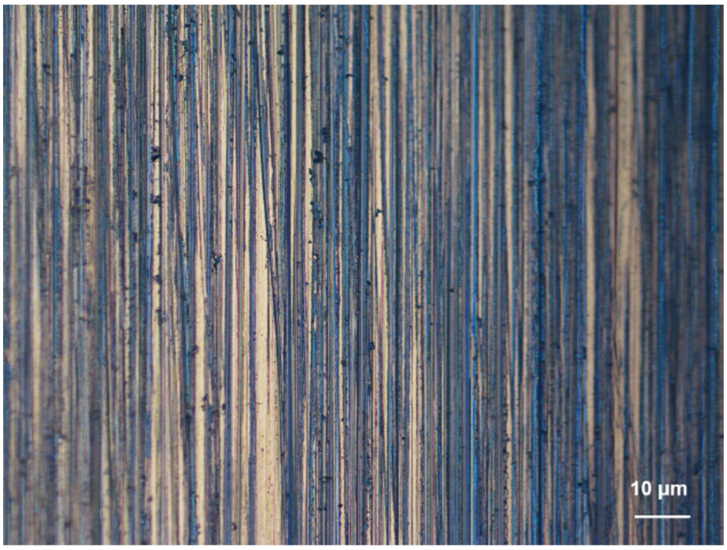


**Figure S1.** The surface microstructure of the Al sheet acquired by an optical microscope.

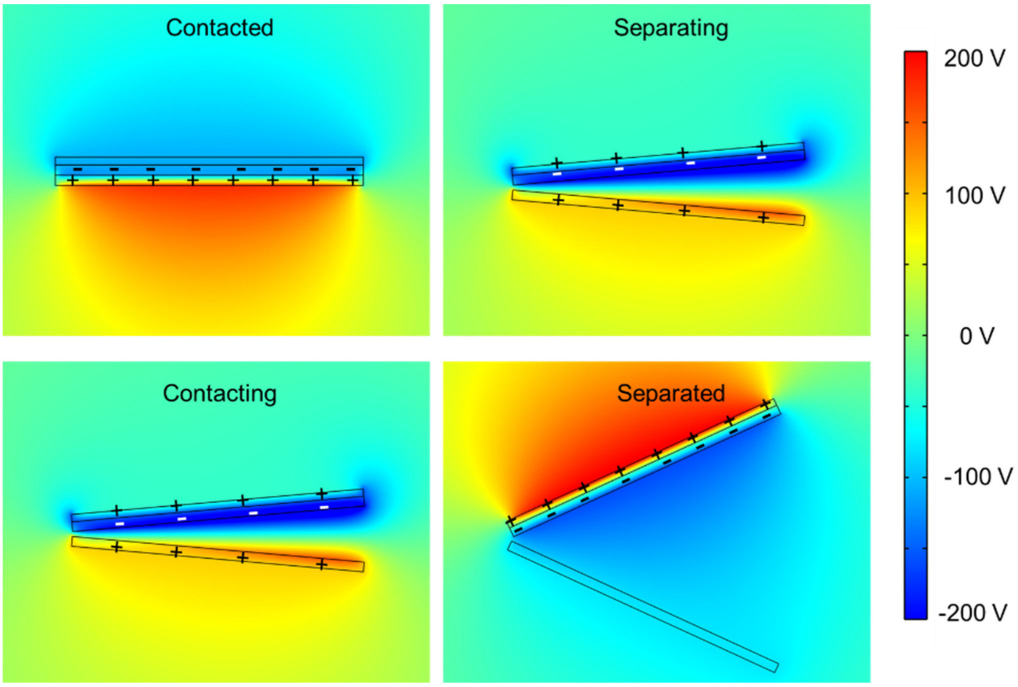


**Figure S2**. The TENG's electrical distribution of charges with COMSOL software.

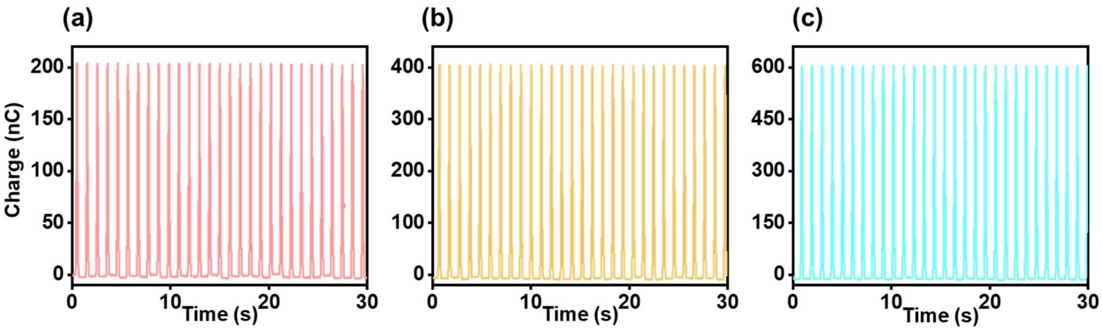


**Figure S3**. (**a**) The $Q_{sc}$ when two PET layers working. (**b**) The $Q_{sc}$ when three PET layers working. (**c**) The $Q_{sc}$ when four PET layers working.

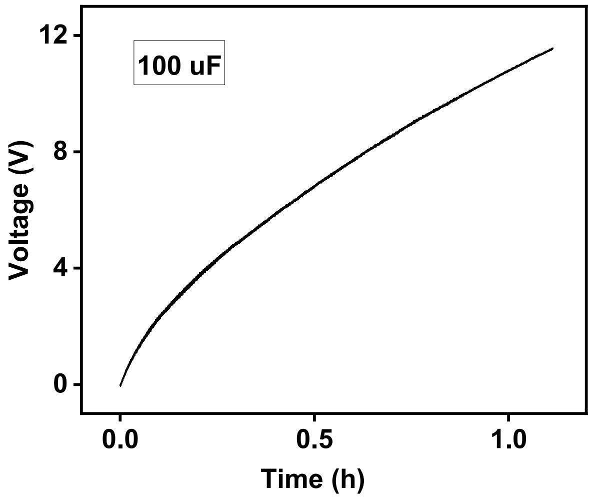


**Figure S4**. The charging curve of the TENG.

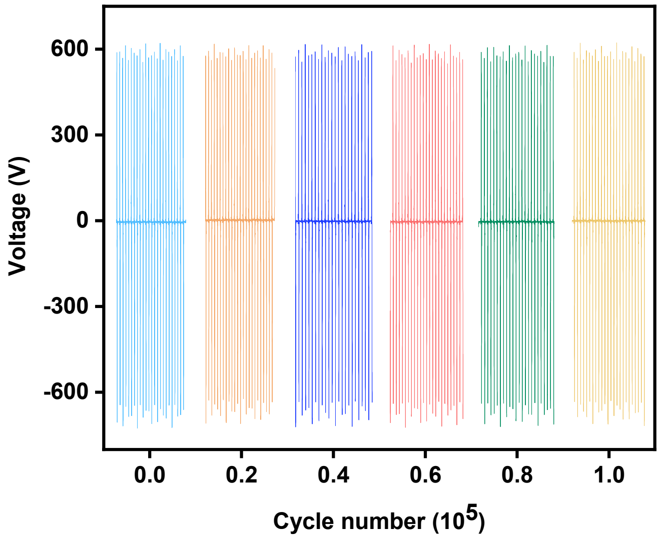


**Figure S5.** The stability test of the TENG.

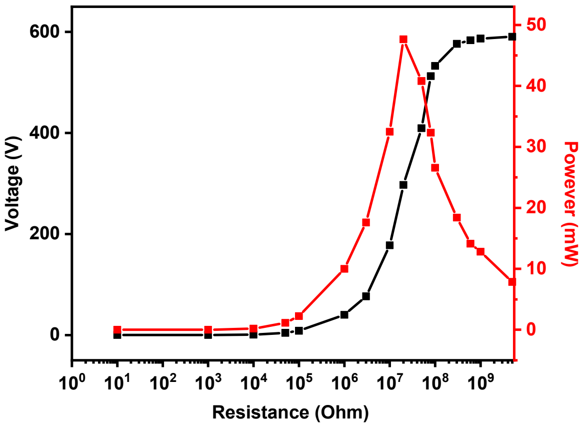


**Figure S6**. The power measurement of the TENG.

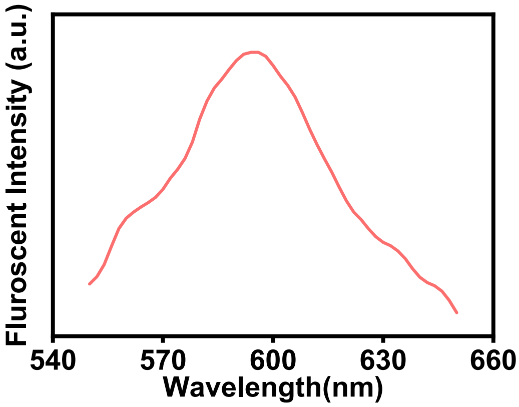


**Figure S7**. The fluorescence emission curve of the DOX.

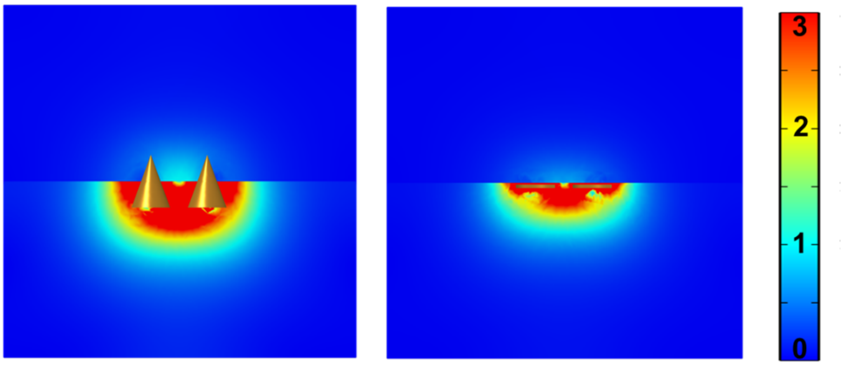

**Figure S8**. The finite element analysis of the microneedle electrode and planar electrode with the voltage of 200 V. EF: KV/cm.

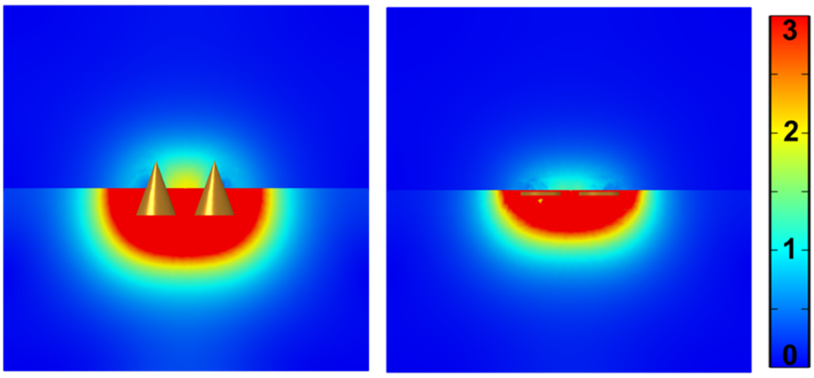


**Figure S9**. The finite element analysis of the microneedle electrode and planar electrode with the voltage of 400 V. EF: KV/cm.

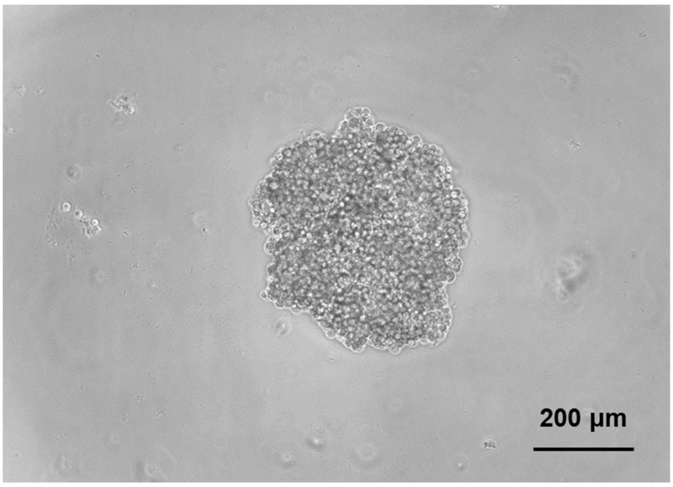


**Figure S10**. The optical microscope image of the MCTS on Day 0.
